# Cross-wavelet analysis allows obtaining high temporal and frequency resolution in heart rate synchrony analysis

**DOI:** 10.3389/fnetp.2026.1869004

**Published:** 2026-07-08

**Authors:** Bernadette F. Denk, Stella Wienhold, Nina Volkmer, Nikolaus F. Troje, Maria Meier, Jens C. Pruessner

**Affiliations:** 1 Department of Psychology, University of Konstanz, Konstanz, Germany; 2 Centre for the Advanced Study of Collective Behaviour, University of Konstanz, Konstanz, Germany; 3 Department of Biology, Centre for Vision Research, York University, Toronto, ON, Canada; 4 Department of Child and Adolescent Psychiatry/Psychotherapy, University of Ulm, Ulm, Germany

**Keywords:** autonomic nervous system, cross-correlation, cross-wavelet power, interpersonal synchrony, network physiology, social interaction, synchrony methods, time-frequency analysis

## Abstract

Interpersonal synchrony, the temporal correspondence of repeated behavioral, physiological, or neural measures between individuals, has been analyzed using a variety of different methods. However, the analysis method needs to be carefully chosen to ensure an adequate interpretation of the results. Here, we suggest using cross-wavelet power for interpersonal synchrony analysis due to several advantages. We demonstrate the proposed method using the example of heart rate synchrony. In cross-wavelet power analysis, synchrony is determined per frequency band and time point, allowing for both fine-grained frequency and temporal resolution. We argue that applying this approach to analyze synchrony in heart rate data provides additional information about underlying processes that may influence overall heart rate alignment between individuals. We describe the principles of cross-wavelet power analysis and compare the method to the frequently used cross-correlational approach using simulated and real data. Cross-correlation is a linear measure of the similarity between time series, which includes the quantification of leader-follower relationships. We illustrate different implications for which data series are considered to be synchronous and describe the advantages and drawbacks of using cross-wavelet analysis across various possible synchronization scenarios. The main advantage of cross-wavelet power is its high time- and frequency resolution, whereas cross-correlation is more suitable when researchers aim to differentiate between synchrony types. Finally, we provide recommendations for implementing cross-wavelet power analysis, including R code to facilitate the application to one’s own data.

## 1 Interpersonal synchrony and its quantification

Interpersonal synchrony, defined as the correspondence between two or more individuals’ trajectories, has been increasingly discussed in the literature (daSilva and Wood, 2024; Mayo et al., 2021; Hoehl et al., 2021). In a network physiology context, interpersonal synchrony can be conceptualized as an example of dynamical interactions across systems (in this case, between individuals), where the linkage between systems and its quantification is in focus (Bashan et al., 2012). Synchrony has been observed in diverse modalities, including behavior, movement, facial expressions, autonomic nervous system activity, endocrine trajectories, cognition, and emotions (Shamay-Tsoory et al., 2019; daSilva and Wood, 2024; Palumbo et al., 2017). Synchrony can fulfill a range of different functions (daSilva and Wood, 2024), from enhancing interpersonal cohesion and cooperation (Launay et al., 2016) to regulating an infant’s emotions (Atzil and Gendron, 2017). However, synchronizing is not always adaptive; for example, higher physiological synchrony during conflict situations is associated with dysfunctional relationship dynamics (Timmons et al., 2015; Coutinho et al., 2021). Across and even within situations, synchrony can change over time, driven by the emergence of “push” and “pull” factors that align or diverge interaction partners (Gordon et al., 2025). Further research is necessary to deepen our understanding of when synchrony emerges, what function it serves in a given situation, and whether this is adaptive with respect to a specific goal, such as relationship quality or performance (Mayo et al., 2021; Mayo and Gordon, 2020). As of yet, there are open questions regarding what constitutes interpersonal synchrony, and how it can be quantified (Mayo et al., 2021; Palumbo et al., 2017; Gordon et al., 2025; Hoehl et al., 2021). Here, we argue for the use of *cross-wavelet power (CWP) analysis* to quantify interpersonal synchrony. CWP is a time-frequency analysis approach determining the similarity between two time series (i.e., repeated measures). As we will detail below, each time series is first decomposed into its frequency components over time. The overlap of two deconstructed time series constitutes the synchrony measurement (Torrence and Compo, 1998). We aim to introduce the method to synchrony researchers, and discuss when its use is appropriate and which characteristics of the synchronization process it targets. We hope to enable an informed choice of whether the method may be beneficial, depending on the data at hand. CWP has previously been suggested for assessing social interactions, including synchronization patterns (Issartel et al., 2015). Here, we apply the method to the field of physiological synchrony, focusing on interpersonal heart rate synchrony.

We focus on heart rate as an unobtrusive measure of autonomic nervous system activation (Nelson et al., 2020). Specifically, the heart is innervated by two main branches of the autonomic nervous system, the parasympathetic and sympathetic branches (Wehrwein et al., 2016). The autonomic nervous system is involved in diverse regulatory processes, including metabolism and temperature regulation, but also affective responses, emotion regulation, social processes, and stress and relaxation (Wehrwein et al., 2016; Porges, 2003; Ulrich-Lai and Herman, 2009; Beauchaine, 2015). This makes the autonomic nervous system a prime target for investigating interpersonal processes, including autonomic synchronization (Palumbo et al., 2017). Within the autonomic nervous system, the parasympathetic branch is related to social-emotional functioning (Porges, 2003; West and Mendes, 2023), whereas the sympathetic branch is associated with activation, arousal, and stress (Wehrwein et al., 2016; Ulrich-Lai and Herman, 2009). Across both branches, measuring autonomic synchrony can provide valuable insights into interpersonal dynamics (Palumbo et al., 2017), which is why heart rate is a frequently investigated biomarker in interpersonal synchrony research (e.g., Coutinho et al., 2021; Danyluck and Page-Gould, 2019). We propose that analyzing heart rate synchrony using the CWP method allows for a detailed assessment of interpersonal social-emotional trajectories. While the CWP method may also be applied to other autonomic nervous system markers, we focus on heart rate, which is influenced by both autonomic branches. As we will argue, the CWP method may be especially useful for measures such as heart rate, which consist of different underlying processes, i.e., different time scales.

In the past, various approaches have been used to quantify synchrony. While various methods for quantifying synchrony are available (Cliff et al., 2023), they can yield diverging results for the same data and should be carefully chosen based on the data’s characteristics (daSilva and Wood, 2024). Yet, the choice of method is seldom explicitly justified in the literature. Among these, correlational approaches are popular, especially *cross-correlation* (Altmann et al., 2022; Dean and Dunsmuir, 2016), and *actor-partner interdependence models* (Thorson et al., 2018). Both cross-correlation and actor-partner interdependence models quantify the linear relationships between individuals’ data series. Beyond the aforementioned methods, other methodologies are available that extend synchrony analysis to additionally include nonlinear features that may better reflect physiological processes, such as heart rate (Coco et al., 2021; Banerjee and Mitra, 2014). This includes CWP, a nonlinear method that may uncover distinct properties of synchronization processes compared to cross-correlation and actor-partner interdependence models (Quer et al., 2016; Schizas et al., 2025). Other nonlinear synchrony methods include *cross-recurrence quantification analysis* and the *Kuramoto model of synchrony* (Coco et al., 2021; Acebrón et al., 2005). However, rather than some methods being better suited to quantify synchrony, the applicability of each method depends on the data’s underlying properties and aims of the research (daSilva and Wood, 2024). To illustrate how the choice of method impacts the quantification of synchrony in the same data, we compare our method of choice, CWP, to cross-correlation, one of the most widely used methods for synchrony analysis. Specifically, we utilize the R package SUSY (Tschacher, 2022), which provides an easy implementation of windowed cross-correlation. In this way, we highlight CWP’s advantages and disadvantages, and enable an informed choice of synchrony analysis method.

## 2 Types of synchrony

**FIGURE 1:**
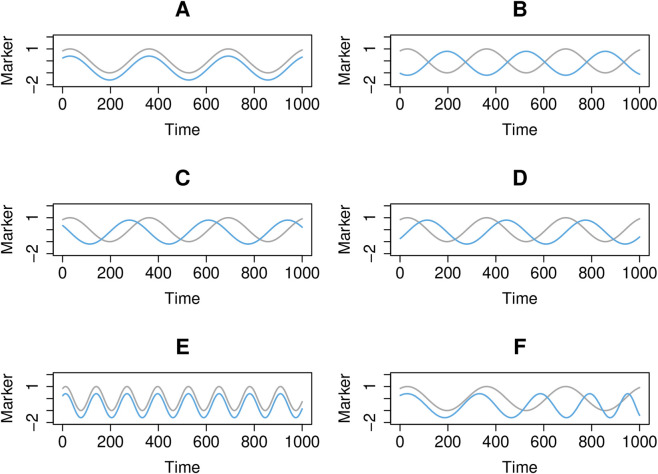
Sine wave data to demonstrate different types of synchrony relevant for the analysis of physiological data from dyadic interactions. Grey and blue lines represent two individuals’ synchronized trajectories. **(A)** In-phase synchrony. **(B)** Anti-phase synchrony. **(C)** Shifted synchrony, with trajectory one leading. **(D)** Shifted synchrony, with trajectory one following. **(E)** In-phase synchrony of higher-frequency sine waves compared to **(A)**. **(F)** Change from in-phase synchrony to desynchronized trajectories.

CWP is a nonlinear method that, broadly speaking, quantifies correspondences and (delayed) influences between time series (Issartel et al., 2015). This means that CWP allows for the identification of several different forms of synchrony processes. Our working definition of synchrony for the illustration of the CWP method is the temporal correspondence between time series, i.e., repeated measures. While open questions remain regarding whether synchrony requires a specific duration or temporality, requires active adaptation of one interaction partner, and whether synchrony can be cross-modal (Hoehl et al., 2021), we do not include these considerations in our data simulation process, and instead define all types of correspondences as synchronous. In Figure 1, we illustrate these different forms using the example of simplified sine wave functions that could represent any biological or psychological marker. These functions represent time series, i.e., repeated measures, which are a prerequisite for identifying correspondences over time, as per our synchrony definition.

Each panel in this figure shows a type of “synchronization” between the grey and blue lines. Figure 1A is an example for *in-phase* synchrony, where changes occur simultaneously and in the same direction. For example, during play, children may experience simultaneous increases and decreases in their heart rate, resulting in in-phase synchrony. Figure 1B depicts *anti-phase* synchrony, i.e., simultaneous changes in opposite directions. This type of synchrony may indicate coregulation; for example, during social interaction, one partner’s breathing rate and heart rate may increase due to heightened arousal, while the other may remain calm and lower their breathing rate, resulting in anti-phase synchrony. Both in-phase and anti-phase synchrony can additionally be *shifted*, i.e., one person’s trajectory changes before the other’s (see Figures 1C,D). The difference between panels C and D lies in which time series is “leading” or “following” the other. Leader-follower relationships may, for example, originate from emotional contagion, which occurs when one person’s emotional state influences another’s (Prochazkova and Kret, 2017). As we will demonstrate below, CWP analysis can detect synchrony for each of these synchrony types.

Beyond these basic synchrony types, two additional considerations illustrate the advantages of CWP analysis compared to other methods. Firstly, the time series in Figures 1A–D consists of sine waves with a specific *frequency*, i.e., a specific number of oscillations per time unit (e.g., seconds). This frequency may differ across situations and variables (for example, changes in heart rate occur more quickly than changes in hormone levels). Figure 1E shows an in-phase synchronization pattern, but with faster oscillations than in Figure 1A. Thus, synchronization takes place on different time scales. Secondly, synchronization may not be consistent across or even within social interactions (Gordon et al., 2025; daSilva and Wood, 2024). Therefore, identifying temporal changes in synchronization is vital to determine the influence of situational characteristics on momentary synchrony (Konvalinka et al., 2011). Figure 1F shows a change in synchrony, during which time series become desynchronized. We will demonstrate how these different types of synchrony (in-phase, anti-phase, shifted, and differences in frequencies and over time) are represented in the CWP analysis framework.

## 3 Principles of cross-wavelet power analysis

To understand how different types of synchrony are present in CWP outcomes, we will first introduce the basic principles of CWP analysis and then illustrate the respective outcomes across synchrony scenarios. With the mathematical notation underlying CWP analysis described in detail elsewhere (e.g., Veleda et al., 2012), we here summarize the basic principles for synchrony researchers.

In a first step, suitable data is needed to conduct the CWP analysis. The method of CWP analysis makes only few assumptions about the data structure. Data is expected to be continuously measured and to contain periodic changes in the specified frequency ranges. In the context of heart rate, periodic ranges between 2 and 32 s include the traditional high- and low-frequency ranges (Shaffer and Ginsberg, 2017). While there is, *per se*, no requirement for the length of the recording and the number of measures, they need to be sufficient to capture the assumed periodic changes over time (De Moortel et al., 2002). While other methods (e.g., cross-correlation, Fourier transform coherence) often require stationary data (Lachaux et al., 2002; Dean and Dunsmuir, 2016), this is not the case for CWP, which can accommodate trends (Lachaux et al., 2002). This allows for the analysis of data that includes a change in its mean over time, e.g., an increase in heart rate in response to stress.

**FIGURE 2:**
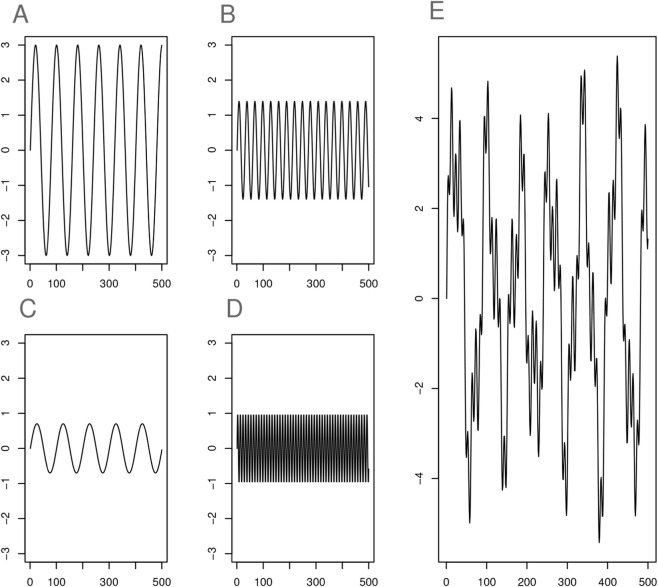
Example of a time series **(E)** consisting of sine waves with different frequencies **(A–D)**. The time series shown in E, resembling real-life physiological data, is constructed by adding the sine waves in A-D. **(A)** Sine wave with high amplitude and low frequency. **(B)** Sine wave with medium amplitude and high frequency. **(C)** Sine wave with low amplitude and low frequency. **(D)** Sine wave with low amplitude and very high frequency. Fourier analysis extracts the power (amplitude) of each constituent sine wave to determine which frequency influenced the resulting time series **(E)** most.

If such data are available, each individual’s data series is first transformed into its frequency components using *wavelet transform* before synchrony with an interaction partner is calculated. Wavelet transform follows a logic similar to the more widely known Fourier transform (Lachaux et al., 2002). In both cases, time series are assumed to consist of underlying oscillatory components across different frequencies (Torrence and Compo, 1998). As an illustrative example, Figures 2A–D shows sine waves with different frequencies and amplitudes. Their addition results in the relatively more complex time series in Figure 2E, which resembles real-life heart rate data. Conversely, Fourier transform can be used to decompose time series E into its constituent sine wave components. Here, Fourier transform not only identifies the frequencies of these components (in this case, four different frequencies), but also their relative influence on the overall course (their *power*). Analogously, the underlying oscillatory components of various time series may be estimated using Fourier transform, including heart rate time series (Quigley et al., 2024). A similar principle underlies wavelet transform. However, while Fourier transform results in the quantification of frequency components across the entire time series, wavelet transform quantifies frequency components for each time point and can thus identify changes over time.

**FIGURE 3:**
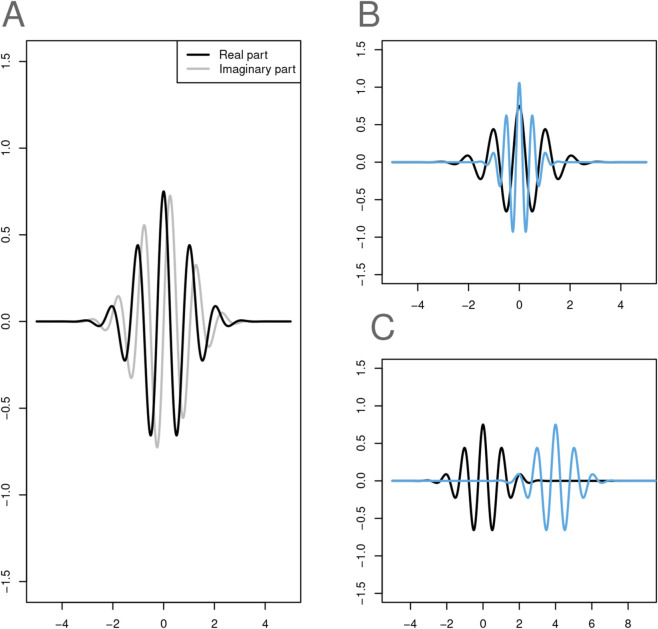
**(A)** The Morlet wavelet function as implemented in (Rösch and Schmidbauer, 2025). **(B)** The blue line depicts a compressed version of the Morlet wavelet function, i.e., a daughter wavelet with a higher frequency, which allows for the identification of higher-frequency components in data. **(C)** The blue line is a time-shifted version of the Morlet wavelet function, which allows for the assessment of time dynamics.

In Fourier transform, the sine waves are referred to as *kernels*. Wavelet transform uses a different kernel, i.e., a wavelet function (Lachaux et al., 2002). Typically, the Morlet wavelet is used, see Figure 3A. This specific wavelet is unique in that it consists of complex numbers, i.e., numbers with a real and an imaginary part (Banerjee and Mitra, 2014; Torrence and Compo, 1998). Stretched and compacted versions of the wavelet function (*daughter wavelets* of the original *mother wavelet*) represent different frequency bands (Figure 3B; Torrence and Compo, 1998). The mother wavelet is the original function from which each daughter wavelet is derived. As with the sine waves in Figure 2, specific frequencies can have greater influence on the overall time series; the signal resembles them more, i.e., they have a greater *power*. In contrast to the sine waves, the wavelet kernel is localized in frequency and time (Lachaux et al., 2002). The power of each daughter wavelet is determined only where the wavelet function is nonzero (i.e., at a specific time point). To assess the power at the next time point, the daughter wavelet is shifted in time by one time unit (Figure 3C). By determining the power of each possible daughter wavelet (one for each frequency and time), the original time series f(t) is transformed into $f_{n}^{j}$ (Veleda et al., 2012). While the original function describes the one-dimensional time series for continuous time points t , the wavelet-transformed function becomes two-dimensional with one data point for each frequency j and each observation n ( n denotes the index of the observation, whereas t denotes, e.g., seconds; Veleda et al., 2012). For the definition of $f_{n}^{j}$ , see the Supplementary Material S1.1. Thus, the wavelet transform converts the original time series into a representation that includes a power coefficient for each time point and frequency band. This reveals how different frequencies influence the series over time.

**FIGURE 4:**
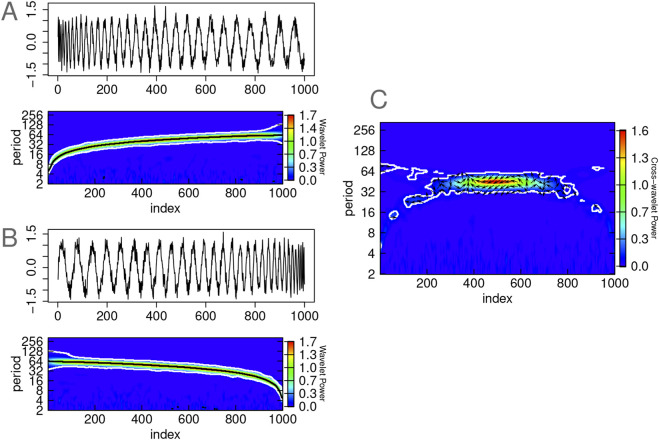
Illustrations of synchrony analysis using CWP. **(A)** A wavelet transform plot showing a periodic data series decreasing in frequency, with added noise. **(B)** A second wavelet transform plot showing a periodic data series increasing in frequency with added noise. **(C)** Visualization of CWP-based synchrony using the overlap in frequencies between both periodic data series over time. The x-axis shows time, and the y-axis depicts the period.

Figure 4 illustrates the wavelet transform process. Figure 4A and B show examples of a simulated time series and its wavelet-transformed counterpart. Each time series was modeled to either increase or decrease in its frequencies (adapted from Rösch and Schmidbauer, 2018). The respective plot below the example time series shows the two-dimensional function $f_{n}^{j}$ of the time series (implemented by the R package WaveletComp; Rösch and Schmidbauer, 2025). The x-axis depicts the time, i.e., the observation index n . Frequency bands j are depicted on the y-axis, here shown as the *period* (period = 1/ j , such that higher frequencies are placed lower on the y-axis; note that the period scale is logarithmic). Colors indicate the relative power of each frequency band over time, with warmer colors representing more power. In this way, changes in frequency components over time become visible. In Figure 4A, the wavelet transform plot indicates that the frequency in the original time series decreases over time (i.e., an increase in period from 4 to 64). Conversely, Figure 4B shows an increase in frequency over time (a decrease in period length from period 64 to period 4). Due to the modeling used to create the time series, only one dominant frequency is present at a time–other frequency bands are colored blue to indicate no systematic influence from these frequencies at that time.

For synchrony analyses, i.e., CWP, two time series are initially each wavelet-transformed separately. CWP is then defined as the similarity between the wavelet-transformed time series at each time point and frequency band (Banerjee and Mitra, 2014; Torrence and Compo, 1998, for the definition of CWP, see the Supplementary Material S1.2). Figure 4C shows the resulting CWP from time series A and B, as implemented in the R package WaveletComp (Rösch and Schmidbauer, 2025). The white line indicates areas of statistically significant synchronization. In our example, the time series overlap in their frequencies at some time points but not others, with the midpoint between start and end resulting in the largest similarity in values (here defined as synchrony). CWP is calculated similarly to a multiplication of two wavelet-transformed time series, where large wavelet-transform values in both time series result in a large CWP synchronization coefficient. In contrast, low values in both or one time series result in low synchrony outcomes. Importantly, this overlap includes not only cases in which frequency components correspond at the exact same time point n , but also correspondence with surrounding time points, e. g., n±10 . Specifically, the width of the daughter wavelet determines how many time points are included in the synchrony calculation at time point n – the wider the daughter wavelet, the more surrounding time points are included.

In summary, CWP first requires a wavelet transform of each time series to quantify the relative impact of specific frequency bands. Then, the overlap between the wavelet-transformed time series is determined, resulting in a synchrony value for each time point and frequency band. As discussed below, this calculation of CWP as the overlap in wavelet-transformed time series has implications for its interpretation as a synchrony measure.

## 4 Synchrony analysis with cross-wavelet power compared to cross-correlation

CWP defines synchrony as the similarity between wavelet-transformed time series. According to this definition, time series are synchronized as long as they overlap in their constituent frequency components. This overlap is analogous to a co-variation between the wavelet-transformed time series (Rösch and Schmidbauer, 2018), which also includes overlap with (some) surrounding time points, i.e., shifted synchrony. To illustrate what this means for synchrony outcomes, we utilized simulated data representing different synchrony scenarios. We also compared the CWP outcomes across scenarios to cross-correlation results. Cross-correlation defines synchrony as the linear relationship between time series. Here, cross-correlation includes not only the relationship between simultaneously measured data points, but also the ability to predict one time series from the other (for a definition of cross-correlation, see the Supplementary Material S1.3). Due to different conceptualizations of synchrony, outcomes may differ between the two methods.

**FIGURE 5:**
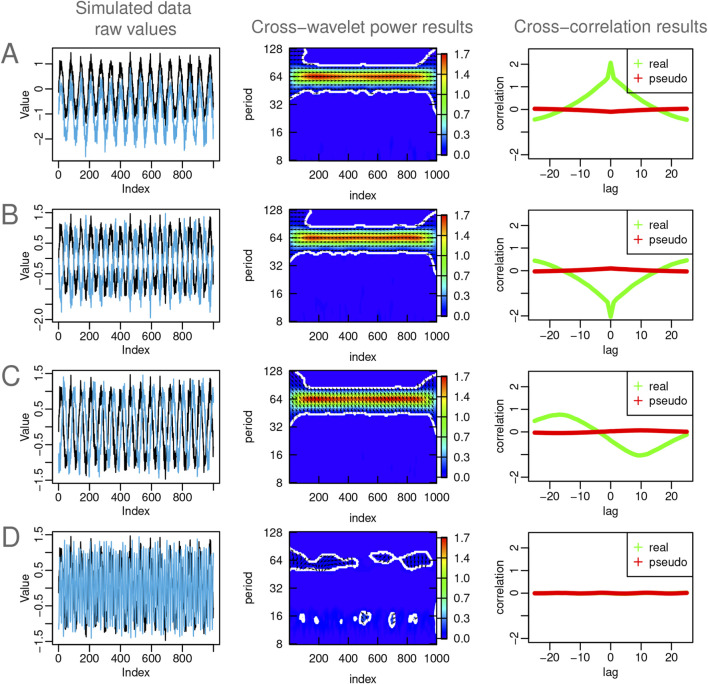
Simulated time series (left column) with corresponding CWP (middle column) and cross-correlation results (right column). **(A)** In-phase synchrony. **(B)** Anti-phase synchrony. **(C)** Shifted synchrony. **(D)** No synchrony. Note that while significant CWP coefficients were present in this scenario, the power values for the cross-wavelet plot were much lower compared to examples where synchrony was present.

To illustrate the outcomes of CWP and cross-correlational analysis for different synchrony scenarios (see Figure 5), we considered in-phase, anti-phase, and shifted synchrony (analogous to Figures 1A–D) and compared the results to non-synchronized time series. In a second step, we added synchrony scenarios including changes in synchrony over time and frequency bands (analogous to Figures 1E,F). We simulated the different synchrony types as periodic time series (via the function periodic. series in the R package WaveletComp; Rösch and Schmidbauer, 2018; 2025). A base time series $f_{1}\left(t\right)$ with a frequency of $\frac{1}{64}$ Hz was used to model different synchrony scenarios. In-phase synchrony (Figure 5A) was modeled via a mean-shift of the base time series $\left(f_{1}\left(t\right)-0.9\right)$ . Anti-phase synchrony (Figure 5B) was modeled by changing the sign of the base time series $\left(-f_{1}\left(t\right)\right)$ . Finally, shifted synchrony (Figure 5C) originated from a time shift of the base function $\left(f_{1}\left(t-20\right)\right)$ . Additionally, we simulated an unrelated time series (Figure 5D) with a different frequency (period = 15). The middle column of Figure 5 depicts the CWP plots for each respective synchrony type.

The results show that the time series in Figures 5A–C produce higher synchrony values than the unrelated time series in Figure 5D. As can be seen, there are no marked differences between in-phase, anti-phase, and shifted synchrony types (Figures 5A–C), resulting in similar patterns and ranges of CWP coefficients. This illustrates that in CWP, synchrony is conceptualized as the correspondence between time series, independent of synchrony type. If a researcher were, for example, only interested in in-phase synchrony, this information would not be available from the CWP plot alone (however, see our discussion on phase-difference measures below).

By contrast, analyzing the simulated data with a cross-correlational approach reveals how this comparison method’s outcomes differ, providing additional information about the specific type of synchrony. In a cross-correlational analysis, synchrony values are calculated for each shift d between two time series (for specifics of the calculation, see the Supplementary Material S1.3l). This means that a correlational measure (interpreted analogously to a Pearson’s correlation coefficient) is obtained for the time series, where either time series is delayed by d = 1, 2, 3, … , D observations. Positive values indicate in-phase synchrony, whereas negative values imply anti-phase synchrony. If the greatest (absolute) cross-correlation coefficient corresponds to a positive lag size, this means that one interaction partner is leading (i.e., time series one); for a negative lag size, the other partner is leading (i.e., time series two). Thus, the cross-correlation function outcome contains several implications about synchrony types and dynamics (Dean and Dunsmuir, 2016).

For the analysis of the simulated data, we use a windowed cross-correlation function, i.e., the measurement duration is split into time windows. Typically, this window is short compared to the overall duration of the measurement (e.g., a window size of 30 s for a recording of several minutes; Altmann et al., 2022) to ensure stationarity of the data. The analysis is repeated for each time window. Here, we used a window size of 60 observations and a maximum delay of 25 s. In Figure 5, right column, the green lines depict the resulting cross-correlation functions. The cross-correlation coefficient (averaged across all windows) for each delay d is shown on the y-axis, and the delay d is shown on the x-axis. Red lines represent pseudo data, i.e., the order of individual values in each time series was randomized (Tschacher, 2022). Unlike the CWP results, the cross-correlational analysis reveals diverging patterns between synchrony scenarios. In-phase synchrony (Figure 5A) is marked by a positive cross-correlation coefficient at d = 0, whereas anti-phase synchrony (Figure 5B) shows a negative cross-correlation coefficient. For shifted synchrony (Figure 5C), the resulting cross-correlation coefficients show a positive peak at d≈−15 , and a negative peak at d=10 (the function was modeled with a shift of −20). Thus, cross-correlation results indicate not only the presence of synchrony, but also the synchrony type. Conversely, Figure 5D shows that cross-correlation coefficients were not consistently larger than those resulting from pseudo data, i.e., no synchronization.

Analyzing simulated data across synchrony scenarios reveals the different information we can obtain from each analysis method. While both CWP and cross-correlation identified simulated synchrony compared to non-synchronized data, cross-correlation additionally indicated the type of synchrony. Positive cross-correlation coefficients revealed in-phase synchrony, negative coefficients revealed anti-phase synchrony, and peaks at lag sizes other than zero revealed shifted synchrony. In this way, information about the underlying (modeled) processes was readily available from cross-correlation, but not CWP results.

## 5 The relevance of temporal and frequency resolution in synchrony analysis

**FIGURE 6:**
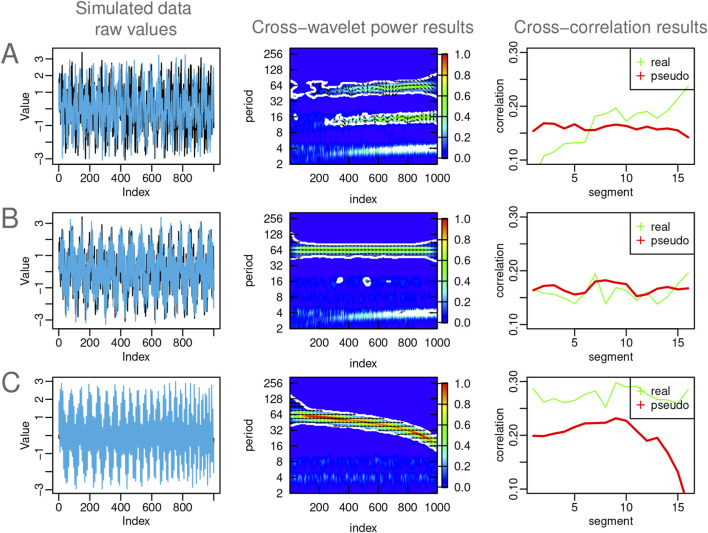
CWP plots (middle column) and cross-correlation results (right column) depicting temporal changes in synchronization patterns of simulated time series (left column). **(A)** Simulated data show a change from no synchrony across frequency bands to synchrony on three different frequency bands. **(B)** Data are simulated to show a change from synchrony on one frequency band to synchrony on two frequency bands. **(C)** Simulated data for a change from synchrony on one frequency band to synchrony on another frequency band.

So far, we have highlighted the advantages of the frequently used cross-correlational analysis–the identification of different synchrony types, i.e., in-phase, anti-phase, and shifted synchrony patterns. However, synchrony in real life situation is unlikely to resemble the simplified cases presented above. We thus simulated additional scenarios, i.e., changes in synchrony over time and across frequency bands. For this, we simulated data based on the time series $f_{1}\left(t\right)$ . Figure 6A depicts two time series that started non-synchronized with increasing synchronization towards the end. The data were modeled to differ in their frequency components at the start and to converge toward the same frequency components at the end (periods 4, 16, and 64). Figure 6 represents a case where synchrony remained constant in one frequency band (period = 64), but additionally emerged in a second frequency band (period = 4) over time. Figure 6C represents a shift in synchrony from a low-frequency band (period = 64) toward a high-frequency band (period = 4). Comparing CWP results (Figure 6, middle column) to cross-correlational results (Figure 6, right column), we see the different representations of these more complex synchrony scenarios.

In Figure 6, middle column, CWP plots depict how synchrony coefficients changed over time and across frequency bands, corresponding to the simulation of the data. In scenario A, synchrony gradually emerged in three distinct frequency bands, as indicated in the corresponding CWP plot. Similarly, the CWP plot for scenario B indicates a gradual emergence of high-frequency synchrony. In scenario C, the CWP plot shows how synchrony shifted from low to high frequencies. The right column shows the results of a windowed cross-correlation analysis. Green lines indicate the absolute cross-correlation coefficient; here, the x-axis does not depict lag size, but the trajectory of absolute synchrony values (independent of lag size) over time, enabling the detection of temporal changes. Again, we chose a window size of 60 and a maximum lag size of 25. Synchrony, as defined by a cross-correlational understanding, was detected to varying degrees. Figure 6A shows that synchrony only developed over time, similarly to the CWP results. In contrast, for the second scenario (Figure 6B), synchrony above chance between the time series was rarely detected. This does not indicate a failure of the cross-correlational method, but rather that the time series were not synchronized in a cross-correlational sense. Finally, synchrony above chance was detected for the time series in Figure 6C, but the simulated change from one frequency band to another is naturally not visible in the cross-correlational results.

The above examples illustrate differences in the processes that are considered synchronous between CWP and cross-correlation analyses. CWP does not distinguish between in-phase, anti-phase, or shifted types of synchrony in its output values (however, see the discussion of phase differences below). Synchrony is instead defined as the covariance between trajectories–depending on the specific frequency band and time point. Synchrony is not conceptualized as an absolute and unchanging measure, but may be restricted to some frequencies and some time points, including time- and frequency-specific changes (Gordon et al., 2025). In contrast, cross-correlation conceptualizes synchrony as the correlation between the *overall* trajectories, not accounting for distinct frequency bands. Additionally, while windowed cross-correlation provides temporal resolution for synchronization results, the window size must be selected carefully in order to adequately capture temporal changes (Altmann et al., 2022).

### 5.1 Application of temporal and frequency resolution in cardiovascular data

With our simulated data, we have demonstrated the frequency-related information available from CWP results. However, the question remains how this information is to be interpreted in real-life applications of CWP analysis. We argue that in the case of heart rate synchrony, obtaining frequency information can meaningfully inform its interpretation.

Heart rate trajectories are controlled by the sympathetic and parasympathetic autonomic nervous system branches (Wehrwein et al., 2016; Gatzke-Kopp et al., 2020). While sympathetic activation increases heart rate via the slower-acting and longer-lasting (nor)adrenaline, parasympathetic changes occur more quickly via faster but shorter-lasting acetylcholinergic influences (Berntson et al., 1993; Thayer and Lane, 2000). These different time scales inform the overall heart rate trajectory depending on the relative activation of both branches. Changes in heart rate that occur quickly (i.e., with a frequency of around 0.15–0.4 Hz = periods 2.5- $6.\bar{6}$ seconds) are likely driven by parasympathetic innervation, whereas a mix of sympathetic and parasympathetic influences underlies slower changes (Shaffer and Ginsberg, 2017; Quigley et al., 2024). The wavelet transform is suitable for extracting the relative power of faster versus slower changes (Quigley et al., 2024), with the power of faster changes being indicative of parasympathetic activation. In the case of dyadic heart rate synchrony, a CWP plot represents the frequency bands on which two individuals are synchronized. Focusing only on synchrony in the frequency bands representing parasympathetic activation (0.15–0.4 Hz = periods 2.5- $6.\bar{6}$ seconds), we can thus quantify the degree to which these individuals’ parasympathetic processes are synchronized. As parasympathetic activation is closely connected to social and emotional processing (Thayer and Lane, 2000; Porges, 2003), synchronization in parasympathetic activation may be relevant for investigating shared social and emotional experiences (Bell, 2020). At the same time, not only parasympatehtic synchrony likely plays a role in social interactions (Palumbo et al., 2017). Thus, synchrony at lower frequencies could indicate distinct relevant autonomic synchrony processes. While the frequency bands associated with parasympathetic activation are certainly of interest in the context of heart rate synchrony, an analysis across the entire relevant frequency spectrum may be warranted to obtain more detailed information about which underlying processes could meaningfully influence synchrony.

For example, if we were to observe a synchrony pattern such as in Figure 6Bi.e., an additional emergence of high-frequency synchrony, this may indicate the emergence of parasympathetic synchrony (i.e., around 0.25 Hz = period 4 s, within the parasympathetic range), while lower-frequency synchrony (outside of this range) would remain constant. Similarly, if we were to observe a synchrony pattern similar to Figure 6C, i.e., a change in synchrony from low to high frequency bands, this could be interpreted as a shift towards synchrony in the parasympathetic frequency range, for example, related to mutual relaxation, emotional processing, or empathy (Palumbo et al., 2017). Indeed, in a previous investigation of 10- to 12-year-olds in an educational group setting, we found that negative affect was related to high-frequency CWP, but not low-frequency CWP (Denk et al., 2026b).

Taking the potentially distinct function of parasympathetic synchrony into account, frequency information may thus provide meaningful information when measuring heart rate synchrony. The same applies to measuring synchrony in other modalities, where assessing frequency information may elucidate the underlying processes that shape the course of synchronization. For example, movement synchrony may occur at different time scales: Interaction partners may synchronize in their hand gestures but also in their overall seating position, with different rhythms of the respective changes in these variables. Frequency ranges included in the analysis should thus be chosen to capture all potentially meaningful processes depending on their respective time scales.

## 6 Recommendations for the implementation

The CWP analysis can be implemented across programming languages. Here, we showed results using the WaveletComp package in R (Rösch and Schmidbauer, 2025). A minimal working example for the implementation is provided in the Supplementary Material S2. Other possibilities include PyCWP in Python (Krieger and Freij, 2025), or the wavelet coherence toolbox in Matlab (Grinsted et al., 2014). These algorithms allow for different degrees of customization, e.g., the use of multiple wavelet functions.

Independent of the algorithmic implementation, there are several considerations regarding the measurement of synchrony and the further processing of the results. To measure synchrony, a baseline should be included, i.e., a period prior to the social interaction during which participants do not interact, while they are individually recorded. Ideally, participants should be separated without the opportunity for auditory or visual contact. This provides a reference value for the CWP analysis against which a later interaction can be (statistically) compared.

After obtaining suitable data, questions remain about how to process the CWP analysis results. CWP matrices can be visualized as images (as shown here); however, this two-dimensional representation may be unsuited for incorporating the results into a statistical model. More likely, a one-dimensional synchrony outcome is required (e.g., for predicting synchrony values in a linear model). Thus, CWP results require aggregation across time and frequency points. Specifically, researchers are required to use an aggregation function, such as the statistical mean, on all values within a specific time and frequency window. For heart rate, a CWP matrix may be segregated into frequencies in and outside the parasympathetic range. Indeed, such a separation may reveal different synchronization processes in a meaningful way (Denk et al., 2026a; b). However, an even more fine-grained analysis of narrower frequency bands could provide additional information (Wienhold et al., 2025). For heart rate synchrony, differentiating upper high-frequency bands (periods 2–4), lower high-frequency bands (periods 4–8), upper low-frequency bands (periods 8–16), and lower low-frequency bands (periods 16–32) may be an appropriate starting point (e.g., Wienhold et al., 2025).

When aggregating CWP coefficients to produce a one-dimensional outcome, another concern is the time window used for the aggregation. The CWP result has the same temporal resolution as the raw measurements (e.g., instantaneous heart rate may be measured once per second). As autocorrelation between subsequent values is typically high, it may be appropriate to aggregate over larger time windows for statistical modeling of synchrony trajectories. For heart rate, synchrony may be aggregated over, e.g., 20, 30, or 60-s windows. This is a compromise that retains sufficient temporal resolution without artificially increasing power through a large number of redundant observations.

To aggregate the data across the chosen frequency and time windows, we suggest using average values or using the maximum per window. Using the maximum value provides information about the highest points of synchrony during the experiment. However, this may lead to overestimating synchrony if there are spuriously large values. Since unified guidelines do not yet exist, a multiverse analysis of different time- and frequency-windows and aggregation methods may be a next step in the investigation of CWP analysis.

Additional considerations in interpreting the results surround the testing against pseudo data, which is often included in synchrony analysis methods such as the cross-correlation analysis implemented by the SUSY package (see Figures 5, 6). The WaveletComp package supports significance testing against pseudo data, with multiple options for simulating the pseudo data (e.g., shuffling the original data or testing against white noise; see Rösch and Schmidbauer, 2018; Rösch and Schmidbauer, 2025). While it may be useful to exclusively include significant synchrony values in one’s analysis, the specific production process of pseudo data may meaningfully alter the results. Additionally, pseudo data may not be a neutral comparison, for example, due to task-specific influences. We therefore recommend testing against a baseline measurement phase of the experiment instead of relying on pseudo data.

While CWP analysis has proven useful in analyzing synchrony (Issartel et al., 2015), open questions remain regarding its implementation. For analyzing synchrony of heart rate trajectories, there are no established guidelines regarding data aggregation. The main recommendation above all other suggestions we made here is that researchers should take into account the characteristics of their study and data to determine the most suitable data processing operations after a CWP analysis is performed.

## 7 Application to real-life data from a social interaction in human dyads

As an extension of our investigation, we applied the CWP method (and the cross-correlation method) to real-life data to showcase more realistic outcome values. For this, we used data collected during an experiment in an undergraduate psychology class. The study was conducted in accordance with the Declaration of Helsinki, and the protocol was approved by the University of Konstanz Ethics Committee. After providing informed consent, we recorded heart rate data from N = 10 participants (mean age = 21.10, SD = 1.29, all female). The participants formed two groups ( n = 10 dyads each), in which we calculated heart rate synchrony for each dyadic combination, resulting in N = 20 dyads. Experimental conditions were counterbalanced such that n = 10 dyads were tested in the same order. We calculated heart rate synchrony across the entire experiment for each dyad.

The experiment aimed to investigate whether heart rate synchrony increased during a group interaction compared to a non-interactive baseline, and whether the degree of synchrony would further depend on the presence or absence of eye contact during the interaction. During the 10-min baseline, participants were sitting apart from each other in the same room. They were instructed to keep themselves occupied quietly while refraining from any social contact. Afterwards, participants split into two groups and played a dice game (“Mäxle”), a type of “Liar’s dice” game, in German. Each group played the game under two experimental conditions for 5 minutes each in a counterbalanced order. The game was played either with eyes open or blindfolded. In the blindfolded condition, participants lifted their blindfolds only briefly to check their dice, but refrained from eye contact with other participants. Following the two interaction tasks, another 10-min baseline was conducted, analogous to the first.

Raw R-R intervals were recorded from Polar H-10 heart rate sensors (sampling rate 1,000 Hz; Polar Electro Oy, Kempele, Finland) using the Heart Rate Variability Logger App (Altini, 2013). The data were outlier-corrected and interpolated to heart rate values with a sampling frequency of 1 Hz. Dyadic synchrony was calculated as CWP using the WaveletComp package (Rösch and Schmidbauer, 2018; Rösch and Schmidbauer, 2025) and as cross-correlational outcomes using the SUSY package (Tschacher, 2022) in R.

**FIGURE 7:**
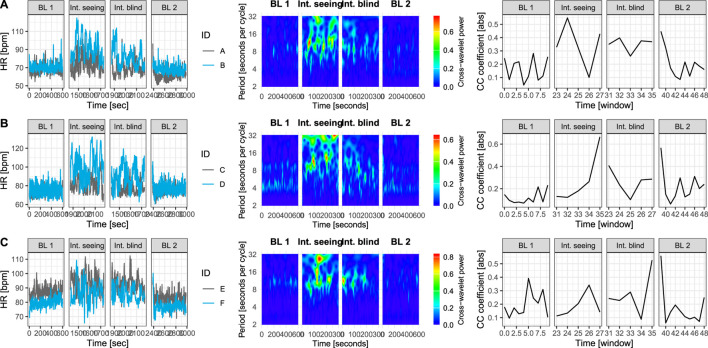
Heart rate data (left column) of three example dyads **(A–C)**, and corresponding CWP (middle column) and cross-correlation (right column; averaged over 60-s windows) outcomes. Note that CWP outcomes are also specific to the frequency band in which the synchronization occurs.

To illustrate how raw heart rate data correspond to synchrony outcomes, Figure 7 shows heart rate trajectories and resulting synchrony outcomes for three different example dyads A–C. The left side depicts the heart rate trajectories of the two dyad members for the two baseline measures (*BL 1* and *BL 2*), and the two game sequences with eyes open (*Int. seeing*) and closed (*Int. blind*). The middle column of Figure 7 shows CWP results for the corresponding experimental phases. The example dyads illustrate what synchrony outcomes may look like for real-life data. For CWP, differences emerged between the frequency bands, suggesting that different heart rate frequencies may have distinct roles.

As described above, CWP outcomes are typically aggregated for further statistical analysis. Here, we selected two distinct frequency bands–periods 4–8 (lower high-frequency) and periods 8–16 (upper low-frequency; see Wienhold et al., 2025). We further averaged CWP coefficients across 60-s time windows. This makes the results comparable to those of the windowed cross-correlation, where we also used 60-s windows (results are depicted in the right column of Figure 7).

**FIGURE 8:**
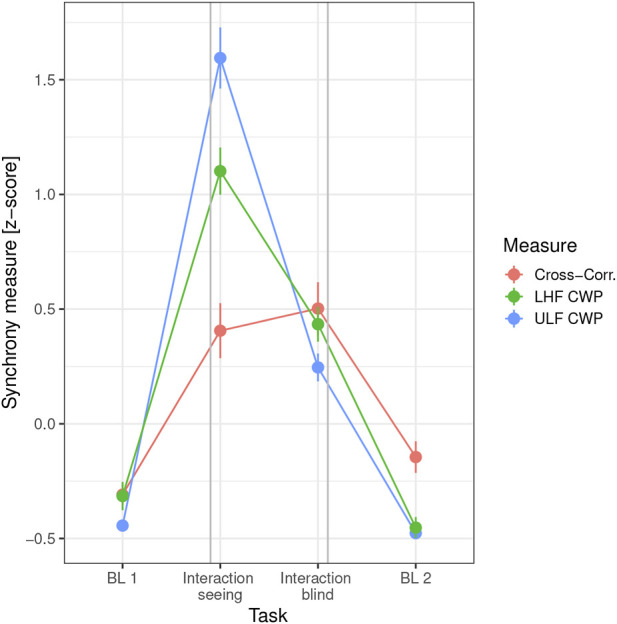
Comparison between z-standardized synchrony measures: lower high-frequency CWP (LHF CWP), upper low-frequency CWP (ULF CWP), and absolute cross-correlation coefficients in real data across experimental phases. Horizontal lines indicate beginning and end of the interaction part of the experiment. Note that only CWP measures reveal the effect of the vision condition.

To explore potential frequency-specific changes in CWP throughout the experiment, we focused on the lower high-frequency (LHF) and upper low-frequency bands (ULF), comparing these outcomes among themselves and to cross-correlation-based results. The LHF range is associated with parasympathetic processes, whereas the ULF range is influenced by both parasympathetic and sympathetic activation (Shaffer and Ginsberg, 2017). Figure 8 compares LHF and ULF CWP with cross-correlation outcomes for the entire sample. Here, we z-standardized all synchrony measures to increase comparability. Three multi-level mixed-effects models (Pinheiro and Bates, 2000) predicting LHF and ULF CWP and cross-correlation, respectively, with the fixed effects window number and experimental phase (reference condition *BL 1*) and random slopes for each dyad showed that CWP and cross-correlation coefficients both increased during the interactions compared to *BL 1* (LHF CWP: $\beta_{\mathrm{seeing}}$ = 2.22, p<.001 ; $\beta_{\mathrm{blind}}$ = 1.57, p<.001 ; ULF CWP: $\beta_{\mathrm{seeing}}$ = 2.26, p<.001 ; $\beta_{\mathrm{blind}}$ = 0.89, p<.001 ; cross-correlation: $\beta_{\mathrm{seeing}}$ = 0.13, p<.001 ; $\beta_{\mathrm{blind}}$ = 0.14, p<.001 ). Additionally, LHF CWP was higher during the second compared to the first baseline (*BL 2* compared to *BL 1*, $\beta_{\mathrm{BL2}}$ = 1.13, p = 0.002). These results indicate that all three synchrony measures varied significantly throughout the course of the experiment, with higher synchrony during interaction phases compared to a non-interactive baseline.

Next, we compared the different outcome trajectories. A multi-level mixed effects model including all three synchrony measures (z-standardized) and an interaction effect between *synchrony method* (reference condition: *cross-correlation*) and *experimental phase* showed a significant positive interaction effect for LHF CWP ( β = 0.72, p<.001 ) and ULF CWP ( β = 1.34, p<.001 ) during the *seeing interaction* phase. Additionally, LHF CWP was lower during the *BL 2* experimental phase, compared to the reference conditions *cross-correlation* and *BL 1*. Thus, compared to cross-correlation, CWP allowed for more detailed information on the dynamics occurring during the different experimental phases and may be more sensitive to changes in social interactions. An analogous model that compared only the two CWP measures revealed a positive interaction between ULF CWP and the *seeing interaction* ( β = 0.622, p<.001 ) compared to the reference levels LHF CWP and *BL 1*. This indicates that even within the CWP method, different frequency bands may be associated with specific interpersonal processes. In our example data, parasympathetic cardiac synchrony (LHF CWP) showed a significantly distinct pattern from lower-frequency synchrony bands (ULF CWP, see Figure 8).

Real-life data illustrate the distinct synchrony patterns resulting from different analysis methods. While all synchrony indicators showed increased synchrony during the interaction phases, CWP measures additionally reflected changes between the *seeing* and *blind* experimental conditions. Even within CWP measures, low-frequency synchrony increased more during the interaction than high-frequency, parasympathetically driven synchrony. This points to distinct roles of different oscillatory patterns within the heart rate synchronization process. Depending on the specific research question, researchers may be interested in overall synchrony, which could be measured with both cross-correlation and CWP, or in the distinct processes underlying synchrony, which could be captured via the CWP method.

## 8 Discussion

While synchrony, including autonomic nervous system synchrony, is a promising avenue for investigating interpersonal interactions (Palumbo et al., 2017; Mayo et al., 2021), its quantification is subject to ongoing discussions. Synchrony can take on different forms, i.e., in-phase versus anti-phase and concurrent versus shifted synchronization processes. In addition to these more “traditional” approaches to differentiating synchrony, other considerations include dynamic changes in synchrony or different frequencies at which synchrony occurs (Gordon et al., 2025). Here, we applied CWP analysis to demonstrate its utility, particularly in the latter cases, i.e., the changes of synchrony across frequency bands over time. Due to its high time- and frequency resolution, CWP analysis can detect such changes when they occur in the data (Quer et al., 2016).

### 8.1 Advantages of cross-wavelet power analysis

Analyzing synchrony using a CWP approach enables researchers to obtain results with a high time- and frequency-resolution. A temporally resolved depiction of synchrony conceptualizes synchrony not as a static characteristic of interaction but as a dynamically fluctuating process. Increasingly, synchrony is understood to change dynamically during social interactions (Mayo and Gordon, 2020; Gordon et al., 2025). While it is generally understood that increased synchrony is not always “better” in terms of interpersonal or goal-directed outcomes (Timmons et al., 2015; Coutinho et al., 2021), recent discussions have added the notion of the adaptivity of within-situation fluctuations of synchrony. To assess these, an analysis method including a high temporal resolution is necessary (Gordon et al., 2025). CWP possesses a temporal resolution as fine-grained as the original measurement. While CWP outcomes, in practice, will often still need to be aggregated across measurement points, the method itself does not impose restrictions on the analysis of temporal fluctuations.

The most striking advantage of CWP, however, is its ability to detect changes in underlying frequencies over time. As discussed above, this is particularly relevant for heart rate synchrony, where frequency bands correspond to specific physiological processes (Shaffer and Ginsberg, 2017). Specifically, high-frequency CWP represents parasympathetic synchrony. As parasympathetic activation relates to multifaceted aspects of socio-emotional functioning (Beauchaine, 2015; Thayer and Lane, 2000), the synchronization between multiple participants’ parasympathetic activity may indicate shared emotional processes (Palumbo et al., 2017; Denk et al., 2026b). While parasympathetic indices and their synchronization can be calculated outside of the CWP framework, CWP offers an additional advantage. The high-frequency bands traditionally considered in parasympathetic measures are broadly applicable approximations of parasympathetic functioning (Shaffer and Ginsberg, 2017). CWP allows us to consider additional relevant frequency bands when examining parasympathetic synchrony. Moreover, frequency bands of interest do not have to be determined in advance; instead, a data-driven approach is possible. This enables assessing the most relevant frequency bands for each dyad. While we focused on heart rate synchrony, frequency information may also be relevant in other synchrony modalities, as changes can occur on different timescales. For example, using CWP for interbrain synchrony analysis may uncover the oscillatory components of brain activity that synchronize between individuals (Nguyen et al., 2021). Together, we saw that CWP offers additional insights into processes underlying overall synchronization.

### 8.2 Disadvantages of cross-wavelet power

Some limitations need to be considered when using CWP to quantify synchrony. Specifically, while the high time- and frequency resolution is advantageous, the two-dimensional result, including thousands of individual data points, often necessitates an aggregation across time points and frequency bands. There are multiple ways to achieve this aggregation. Aggregation techniques potentially influence results, and careful consideration of parameters such as window size is necessary. Thus, using CWP poses the risk of inadequate aggregation of the high-resolution results, yielding suboptimal information about synchrony outcomes. That said, similar issues arise in cross-correlational analyses, where window size and standardization approaches can meaningfully alter the results. (Altmann et al., 2022).

Additionally, one disadvantage of CWP compared to cross-correlation analysis is that CWP coefficients do not indicate the type of synchrony. Cross-correlation coefficients can be positive or negative, indicating in-phase and anti-phase synchrony, respectively. They also include information about the relevant lag sizes, i.e., leader-follower relationships. In contrast, CWP coefficients depict the overall correspondence between time series. This makes CWP results less easily interpretable than other synchrony outcomes. However, CWP analysis provides additional information about synchrony types in the form of phase difference measures (see below). Table 1 summarizes the advantages and disadvantages of the CWP and cross-correlation methods.

**TABLE 1:** Overview of the advantages and disadvantages of cross-wavelet power (CWP) and cross-correlation as methods for quantifying interpersonal (heart rate) synchrony.

​	CWP	Cross-correlation
Advantages	• Time resolution captures dynamical changes during interactions. • Frequency resolution enables differentiating high-frequency from low-frequency synchrony (e.g., separating parasympathetic synchrony in heart rate; a finer resolution is possible)	• Results indicate synchrony type: Positive values indicate in-phase synchrony, negative values indicate anti-phase synchrony, significant coefficients at lag sizes other than 0 indicate shifted synchrony
Disadvantages	• Two-dimensional result requires aggregation for many statistical analyses. • CWP coefficients do not indicate the type of synchrony (may be achieved using phase differences, but no built-in information)	• No temporal resolution (may be achieved by using time windows) • No frequency resolution. In the case of heart rate data, parasympathetic activation needs to be extracted separately (as heart rate variability) to calculate parasympathetic synchrony • Assumption of linear relationship and stationary data

### 8.3 Leader-follower relationships in cross-wavelet power

As discussed, a disadvantage of CWP analysis is a lack of differentiation between potentially divergent processes. In-phase and anti-phase synchrony may reveal distinct interpersonal dynamics (Reed et al., 2013). Moreover, leader-follower relations can provide insights into who adjusts to whom in a social situation. While this information is readily available in cross-correlational outcomes, this is not the case for CWP coefficients. However, in addition to calculating coefficients, we can also extract information about these dynamics within a CWP framework, i.e., by quantifying *phase differences*; however, this is not the focus of the present investigation. For details on phase differences, see the Supplementary Material S3.

The phase difference represents the *argument*, i.e., the angle, of a complex number obtained from the CWP calculation (Rösch and Schmidbauer, 2018). This angle can take values in the interval [−π,π] , representing the difference between the two time series’ phases, i.e., the leader-follower relationship in a dyad (see the Supplementary Material S3 and Aguiar-Conraria and Soares, 2011, for more details). CWP analysis produces a phase difference value at each time and frequency point. The resulting angles can be included in CWP plots (see Figures 5, 6), illustrating not only the absolute magnitude of instantaneous synchrony, but also the synchrony type at that time point.

To exemplify the use of phase difference, we quantified phase difference outcomes from our example data. This analysis is detailed in the Supplementary Material S3. In Figure 5, illustrating CWP outcomes for different synchrony types, we analyzed the phase differences of statistically significant CWP coefficients. We found that for Figure 5A, illustrating in-phase synchrony, all significant phase-difference measures indicated in-phase synchrony, with no measures indicating anti-phase synchrony. Conversely, for the anti-phase synchrony example data (Figure 5B), all phase differences indicated anti-phase synchrony. For the shifted synchrony data (Figure 5C), phase-difference measures indicated shifted synchrony with the same time series leading across the experiment. Additional outcomes of phase-difference measures are available in the Supplementary Material S3 S3. For the simulated data, we thus found that phase differences were consistent with the simulated properties, i.e., consistently indicated in-phase versus anti-phase synchrony, and leader-follower relationships.

In this way, we can mitigate the drawbacks of CWP analysis of unspecific synchronization processes. In CWP analysis, we could differentiate between, e.g., in-phase and anti-phase values as well as assess leader-follower dynamics. Thus, when analyzing synchrony using CWP, researchers can take into account different leader-follower information to a greater or lesser degree.

### 8.4 Future directions and conclusion

Here, we have shown that CWP can be a suitable method for analyzing interpersonal synchrony, especially heart rate synchrony. As of yet, open questions remain regarding its implementation. It remains unclear how CWP results should be aggregated to reduce the number of data points (especially for statistical analysis), while retaining meaningful information about dynamical processes. Moreover, researchers may consider phase difference information in their analysis, e.g., using only in-phase CWP coefficients, or restricting their analysis to statistically significant values (see Supplementary Material S3). Wider adoption and discussion of the method in synchrony research would be beneficial for creating guidelines on appropriate analytical decisions. In general, studies often lack justifications for the methods used to quantify synchrony. Openly discussing why a specific method was chosen would facilitate synchrony research and inspire meaningful exchanges about what constitutes synchrony.

Beyond the context of interpersonal synchrony, the CWP method may also advance research in related topics in network physiology. Specifically, the linkage between physiological systems within an individual requires suitable quantification methods to reveal the network topology (Bashan et al., 2012). While cross-correlational analysis has been used for this quantification (Bashan et al., 2012), physiological time series are generally fractal (Schizas et al., 2025), which may be appropriately modeled using the wavelet transform (Hazewinkel, 1993). Thus, the CWP method may be applied to intraindividual in addition to interpersonal synchronization processes.

From our analysis of simulated and real data, we conclude that CWP may detect meaningful interpersonal processes, such as shared changes from sympathetic to parasympathetic activation. Because of the large amount of information obtained by means of CWP, we believe it to be often superior to cross-correlational analysis when used for physiological data. We hope to inspire synchrony researchers to utilize CWP analysis when appropriate for their specific use case.

## Data Availability

The original contributions presented in the study are included in the article/Supplementary Material. The code for data simulation and analysis is available under https://gitlab.inf.uni-konstanz.de/bernadette.denk/cwp_for_sync_analysis.

## References

[B1] Acebrón J. A. Bonilla L. L. Pérez Vicente C. J. Ritort F. Spigler R. (2005). The Kuramoto model: a simple paradigm for synchronization phenomena. Rev. Mod. Phys. 77, 137–185. 10.1103/RevModPhys.77.137

[B2] Aguiar-Conraria L. Soares M. J. (2011). *The Continuous Wavelet Transform: A Primer*. NIPE Working Papers 16/2011. NIPE - Universidade do Minho.

[B3] Altini M. (2013). Heart rate variability logger (version 5.1.0) (mobile app). Available online at: https://apps.apple.com/de/app/heart-rate-variability-logger/id683984776 (Accessed June 25, 2026).

[B4] Altmann U. Strauss B. Tschacher W. (2022). Cross-Correlation- and entropy-based measures of movement synchrony: non-convergence of measures leads to different associations with depressive symptoms. Entropy 24, 1307. 10.3390/e24091307 36141194 PMC9497848

[B5] Atzil S. Gendron M. (2017). Bio-behavioral synchrony promotes the development of conceptualized emotions. Curr. Opin. Psychol. 17, 162–169. 10.1016/j.copsyc.2017.07.009 28843112 PMC5617801

[B6] Banerjee S. Mitra M. (2014). Application of cross wavelet transform for ECG pattern analysis and classification. IEEE Trans. Instrum. Meas. 63, 326–333. 10.1109/TIM.2013.2279001

[B7] Bashan A. Bartsch R. P. Kantelhardt J. W. Havlin S. Ivanov P. C. (2012). Network physiology reveals relations between network topology and physiological function. Nat. Commun. 3, 702. 10.1038/ncomms1705 22426223 PMC3518900

[B8] Beauchaine T. P. (2015). Respiratory sinus arrhythmia: a transdiagnostic biomarker of emotion dysregulation and psychopathology. Curr. Opin. Psychol. 3, 43–47. 10.1016/j.copsyc.2015.01.017 25866835 PMC4389219

[B9] Bell M. A. (2020). Mother-child behavioral and physiological synchrony. In Advances in Child Development and Behavior (Elsevier), vol. 58. 163–188. 10.1016/bs.acdb.2020.01.006 32169195

[B10] Berntson G. G. Cacioppo J. T. Quigley K. S. (1993). Respiratory sinus arrhythmia: autonomic origins, physiological mechanisms, and psychophysiological implications. Psychophysiology 30, 183–196. 10.1111/j.1469-8986.1993.tb01731.x 8434081

[B11] Cliff O. M. Bryant A. G. Lizier J. T. Tsuchiya N. Fulcher B. D. (2023). Unifying pairwise interactions in complex dynamics. Nat. Comput. 3, 883–893. 10.1038/s43588-023-00519-x 38177751

[B12] Coco M. I. Monster D. Leonardi G. Dale R. Wallot S. (2021). Unidimensional and multidimensional methods for recurrence quantification analysis with crqa. R J. 13, 145. 10.32614/RJ-2021-062

[B13] Coutinho J. Pereira A. Oliveira-Silva P. Meier D. Lourenço V. Tschacher W. (2021). When our hearts beat together: cardiac synchrony as an entry point to understand dyadic co-regulation in couples. Psychophysiology 58, e13739. 10.1111/psyp.13739 33355941

[B14] Danyluck C. Page-Gould E. (2019). Social and physiological context can affect the meaning of physiological synchrony. Sci. Rep. 9, 8222. 10.1038/s41598-019-44667-5 31160690 PMC6547677

[B15] daSilva E. B. Wood A. (2024). How and why people synchronize: an integrated perspective. Personality Soc. Psychol. Rev. 29, 1–29doi. 10.1177/10888683241252036 38770754

[B16] De Moortel I. Hood A. W. Ireland J. (2002). Coronal seismology through wavelet analysis. Astronomy and Astrophysics 381, 311–323. 10.1051/0004-6361:20011659

[B17] Dean R. T. Dunsmuir W. T. M. (2016). Dangers and uses of cross-correlation in analyzing time series in perception, performance, movement, and neuroscience: the importance of constructing transfer function autoregressive models. Behav. Res. Methods 48, 783–802. 10.3758/s13428-015-0611-2 26100765

[B18] Denk B. F. Meier M. Ocklenburg S. Packheiser J. Wienhold S. Volkmer N. (2026a). Coupled hearts – effect of partner stress on cardiac synchronization. Biol. Psychol. 204, 109205. 10.1016/j.biopsycho.2026.109205 41638467

[B19] Denk B. F. Pruessner J. C. Farah S. Barth C. Putra P. U. Lenggenhager B. (2026b). Cardiac synchrony, peer relationships, and affective experiences in children during group interactions. Sci. Rep. 16, 7740. 10.1038/s41598-026-41275-y 41741623 PMC12949066

[B20] Gatzke-Kopp L. M. Benson L. Ryan P. J. Ram N. (2020). Cortical and affective regulation of autonomic coordination. Psychophysiology 57, e13544. 10.1111/psyp.13544 32039482 PMC7324931

[B21] Gordon I. Tomashin A. Mayo O. (2025). A theory of flexible multimodal synchrony. Psychol. Rev. 132, 680–718. 10.1037/rev0000495 39446615

[B22] Grinsted A. Moore C. J. Jevrejeva S. (2014). A Cross Wavelet and Wavelet Coherence Toolbox for MATLAB. MATLAB toolbox. Available online at: http://www.glaciology.net/wavelet-coherence.

[B23] Hazewinkel M. (1993). “Wavelets understand fractals,” in Wavelets: An Elementary Treatment of Theory and Applications (WORLD SCIENTIFIC), 209–219. 10.1142/9789814503747_0012

[B24] Hoehl S. Fairhurst M. Schirmer A. (2021). Interactional synchrony: signals, mechanisms and benefits. Soc. Cognitive Affect. Neurosci. 16, 5–18. 10.1093/scan/nsaa024 32128587 PMC7812629

[B25] Issartel J. Bardainne T. Gaillot P. Marin L. (2015). The relevance of the cross-wavelet transform in the analysis of human interaction - a tutorial. Front. Psychol. 5, 1566. 10.3389/fpsyg.2014.01566 25620949 PMC4288242

[B26] Konvalinka I. Xygalatas D. Bulbulia J. Schjødt U. Jegindø E.-M. Wallot S. (2011). Synchronized arousal between performers and related spectators in a fire-walking ritual. Proc. Natl. Acad. Sci. 108, 8514–8519. 10.1073/pnas.1016955108 21536887 PMC3100954

[B27] Krieger S. Freij N. (2025). PyCWT: wavelet spectral analysis in Python. V. 0.5.0-beta. Python. Available online at: https://github.com/regeirk/pycwt.

[B28] Lachaux J.-P. Lutz A. Rudrauf D. Cosmelli D. Le Van Quyen M. Martinerie J. (2002). Estimating the time-course of coherence between single-trial brain signals: an introduction to wavelet coherence. Neurophysiol. Clinique/Clinical Neurophysiol. 32, 157–174. 10.1016/S0987-7053(02)00301-5 12162182

[B29] Launay J. Tarr B. Dunbar R. I. M. (2016). Synchrony as an adaptive mechanism for large-scale human social bonding. Ethology 122, 779–789. 10.1111/eth.12528

[B30] Mayo O. Gordon I. (2020). In and out of synchrony—behavioral and physiological dynamics of dyadic interpersonal coordination. Psychophysiology 57, e13574. 10.1111/psyp.13574 32221984

[B31] Mayo O. Lavidor M. Gordon I. (2021). Interpersonal autonomic nervous system synchrony and its association to relationship and performance – a systematic review and meta-analysis. Physiol. and Behav. 235, 113391. 10.1016/j.physbeh.2021.113391 33744259

[B32] Nelson B. W. Low C. A. Jacobson N. Areán P. Torous J. Allen N. B. (2020). Guidelines for wrist-worn consumer wearable assessment of heart rate in biobehavioral research. Npj Digit. Med. 3, 90. 10.1038/s41746-020-0297-4 32613085 PMC7320189

[B33] Nguyen T. Hoehl S. Vrtička P. (2021). A guide to parent-child fNIRS hyperscanning data processing and analysis. Sensors 21, 4075. 10.3390/s21124075 34199222 PMC8231828

[B34] Palumbo R. V. Marraccini M. E. Weyandt L. L. Wilder-Smith O. McGee H. A. Liu S. (2017). Interpersonal autonomic physiology: a systematic review of the literature. Personality Soc. Psychol. Rev. 21, 99–141. 10.1177/1088868316628405 26921410

[B35] Pinheiro J. C. Bates D. M. (2000). Mixed-Effects Models in S and S-PLUS. New York: Springer. 10.1007/b98882

[B36] Porges S. W. (2003). Social engagement and attachment: a phylogenetic perspective. Ann. N. Y. Acad. Sci. 1008, 31–47. 10.1196/annals.1301.004 14998870

[B37] Prochazkova E. Kret M. E. (2017). Connecting minds and sharing emotions through mimicry: a neurocognitive model of emotional contagion. Neurosci. and Biobehav. Rev. 80, 99–114. 10.1016/j.neubiorev.2017.05.013 28506927

[B38] Quer G. Daftari J. Rao R. R. (2016). Heart rate wavelet coherence analysis to investigate group entrainment. Pervasive Mob. Comput. 28, 21–34. 10.1016/j.pmcj.2015.09.008

[B39] Quigley K. S. Gianaros P. J. Norman G. J. Jennings J. R. Berntson G. G. De Geus E. J. C. (2024). Publication guidelines for human heart rate and heart rate variability studies in psychophysiology—Part 1: physiological underpinnings and foundations of measurement. Psychophysiology 61, e14604. 10.1111/psyp.14604 38873876 PMC11539922

[B40] Reed R. G. Randall A. K. Post J. H. Butler E. A. (2013). Partner influence and in-phase *versus* anti-phase physiological linkage in romantic couples. Int. J. Psychophysiol. 88, 309–316. 10.1016/j.ijpsycho.2012.08.009 22922526

[B41] Rösch A. Schmidbauer H. (2018). WaveletComp 1.1: a guided tour through the R package

[B42] Rösch A. Schmidbauer H. (2025). Wavelet Comp: computational wavelet analysis. WaveletComp R Package Version 1.2. Available online at: https://CRAN.R-project.org/package=WaveletComp.

[B43] Schizas I. Sullivan S. Kerick S. Mahmoodi K. Cortney Bradford J. Boothe D. L. (2025). Complexity synchronization analysis of neurophysiological data: theory and methods. Front. Netw. Physiol. 5, 1570530. 10.3389/fnetp.2025.1570530 40438270 PMC12116615

[B44] Shaffer F. Ginsberg J. P. (2017). An overview of heart rate variability metrics and norms. Front. Public Health 5, 258. 10.3389/fpubh.2017.00258 29034226 PMC5624990

[B45] Shamay-Tsoory S. G. Saporta N. Marton-Alper I. Z. Gvirts H. Z. (2019). Herding brains: a core neural mechanism for social alignment. Trends Cognitive Sci. 23, 174–186. 10.1016/j.tics.2019.01.002 30679099

[B46] Thayer J. F. Lane R. D. (2000). A model of neurovisceral integration in emotion regulation and dysregulation. J. Affect. Disord. 61, 201–216. 10.1016/S0165-0327(00)00338-4 11163422

[B47] Thorson K. R. West T. V. Mendes W. B. (2018). Measuring physiological influence in dyads: a guide to designing, implementing, and analyzing dyadic physiological studies. Psychol. Methods 23, 595–616. 10.1037/met0000166 29283591

[B48] Timmons A. C. Margolin G. Saxbe D. E. (2015). Physiological linkage in couples and its implications for individual and interpersonal functioning: a literature review. J. Fam. Psychol. 29, 720–731. 10.1037/fam0000115 26147932 PMC4593729

[B49] Torrence C. Compo G. P. (1998). A practical guide to wavelet analysis. Bull. Am. Meteorological Soc. 79, 61–78. 10.1175/1520-0477(1998)079<0061:APGTWA>2.0.CO;2

[B50] Tschacher W. (2022). SUSY: surrogate synchrony. SUSY. R. Package Version 0.1.0. 10.32614/CRAN.package.SUSY

[B51] Ulrich-Lai Y. M. Herman J. P. (2009). Neural regulation of endocrine and autonomic stress responses. Nat. Rev. Neurosci. 10, 397–409. 10.1038/nrn2647 19469025 PMC4240627

[B52] Veleda D. Montagne R. Araujo M. (2012). Cross-wavelet bias corrected by normalizing scales. J. Atmos. Ocean. Technol. 29, 1401–1408. 10.1175/JTECH-D-11-00140.1

[B53] Wehrwein E. A. Orer H. S. Barman S. M. (2016). “Overview of the anatomy, physiology, and pharmacology of the autonomic nervous system,” in Comprehensive Physiology. Terjung R. Editor, 1st edition. (Wiley), 1239–1278. 10.1002/cphy.c150037 27347892

[B54] West T. Mendes W. B. (2023). “Affect contagion: physiologic covariation and linkage offer insight into socially shared thoughts, emotions, and experiences,” in Advances in Experimental Social Psychology (Elsevier), 67, 73–129. 10.1016/bs.aesp.2022.11.002

[B55] Wienhold S. Bär L. Ringleb Z. Zirpel V. Gomolla A. Denk B. F. (2025). The relationship of early life adversity and physiological synchrony within the therapeutic triad in horse-assisted therapy. J. Neural Transm. 132, 1291–1312. 10.1007/s00702-025-02947-7 40423728 PMC12535527

