# Supplementary material for: Cross-wavelet analysis allows obtaining high temporal and frequency resolution in heart rate synchrony analysis
Source: Front Netw Physiol. 2026 Jul 8;6:1869004. doi: 10.3389/fnetp.2026.1869004 (PMC13389767; doi:10.3389/fnetp.2026.1869004)
Supplement: Supplementary file 1 [file Supplementaryfile1.pdf]

Supplementary Material for "Cross-wavelet power analysis allows obtaining high temporal and spatial resolution in physiological synchrony analysis"

1 DEFINITIONS

1.1 Wavelet transform

In the discrete wavelet transform, f_n^j is defined as (adapted from Veleda et al., 2012; Rösch and Schmidbauer, 2018):

$$f_n^j = \int_{-\infty}^{+\infty} f(t)(\psi_n^j)^*(t)dt \tag{S1}$$

Here, ψ_n^j is the Morlet wavelet, localized at frequency j and index n. The original time series $f(t)$ is multiplied by the complex conjugate of this wavelet function, marked by *. Integrating over time points t results in the wavelet-transformed function f_n^j. Conversely, the original $f(t)$ can be approximated by (adapted from Veleda et al., 2012):

$$f(t) \approx \sum_j \sum_n f_n^j \psi_n^j(t) \tag{S2}$$

This equation shows that multiplying the wavelet-transformed function f_n^j with all daughter wavelets ψ_n^j is sufficient to describe the original time series $f(t)$.

1.2 Cross-wavelet power

This similarity, i.e., the cross-wavelet power CWP_n^j, between the wavelet-transformed time series f_n^j and g_n^j, is defined as (adopted from Veleda et al., 2012; Rösch and Schmidbauer, 2018):

$$CWP_n^j \equiv |(2^{j/2} f_n^j) \times (2^{j/2} g_n^j)^*| \tag{S3}$$

Thus, cross-wavelet power is the *modulus* of the *product* of a wavelet-transformed time series with the complex conjugate of another wavelet-transformed time series. The scaling factor $2^{j/2}$, i.e., scaling depending on frequency j, is introduced in some implementations to avoid a systematic underestimation of high-frequency peaks (Veleda et al., 2012).

1.3 Cross-correlation

The cross-correlation coefficient $CC(d)$ for lag d of two time series $f(t)$ and $g(t)$ is calculated as:

$$CC(d) = \frac{\sum_t [(f(t) - m_f)(g(t-d) - m_g)]}{\sqrt{\sum_t (f(t) - m_f)^2} \sqrt{\sum_t (y(t-d) - m_g)^2}} \tag{S4}$$

Here, m_f is the mean of the time series $f(t)$, and m_g is the mean of the time series $g(t)$. In our analysis, we used a windowed cross-correlation function, meaning that only values of $f(t)$ and $g(t)$ within a specific time window are included in the formula.

2 CODE

We present a minimal working example of code in R to conduct a cross-wavelet power analysis in R, using the package WaveletComp (Rösch and Schmidbauer, 2018, 2025). To demonstrate the cross-wavelet power analysis, we simulate data as a periodic time series and include a shift in frequency bands. Raw data are displayed. Next, the cross-wavelet power analysis is conducted, and the results are depicted in a cross-wavelet power plot.

```
library(WaveletComp)
# 1. Create time series:
# Add noise to each time series (newly generated every time)
noise_func <- function(sd = 1){
  0.2*rnorm(1000, sd = sd)
}
# Create two time series with different frequency components
ts1 <- periodic.series(start.period = 2, end.period = 64,
                      length = 1000)+noise_func()
ts2 <- periodic.series(start.period = 64, end.period = 2,
                      length = 1000)+noise_func()
# Plot raw data
plot(ts1, type = "l")
plot(ts2, type = "l")

# Combine into a data frame
df <- data.frame(ts1 = ts1,
                 ts2 = ts2)

# 2. Cross-wavelet power analysis
cwp_ts1ts2 <- analyze.coherency(df, c("ts1", "ts2"))

# Plot result
# Specify color key as either interval ("i") or quantile ("q")
wc.image(cwp_ts1ts2, color.key = "i")
```

3 PHASE DIFFERENCE ANALYSIS

3.1 Phase difference analysis to identify leader-follower relationships

While CWP coefficients do not provide insights into in-phase, anti-phase, or shifted types of synchrony, the CWP analysis can address these questions by complementing the analysis with a *phase difference* measure (Aguiar-Conraria and Soares, 2011). This additional outcome of a wavelet-based analysis uncovers directionality and in-phase versus anti-phase synchronization. To understand this measure, we again consider the calculation of CWP. The multiplication of a Morlet wavelet with a time series results in complex numbers. To calculate CWP coefficients, the modulus of these numbers is used. However, we can make use of another property of complex numbers in analyzing interpersonal synchrony. Specifically, the *argument* of a complex number represents the angle between the real and imaginary dimensions of a complex number (Rösch and Schmidbauer, 2018):

$$\text{Phase}_n^j = Arg((2^{j/2} f_n^j) \times (2^{j/2} g_n^j)^*) \tag{S5}$$

Figure S1. Illustration of the possible values of the phase difference metric. Values indicate in-phase (white) or anti-phase (grey) synchronization, with dyad members x and y leading or following before the other. The arrows represent different example values.

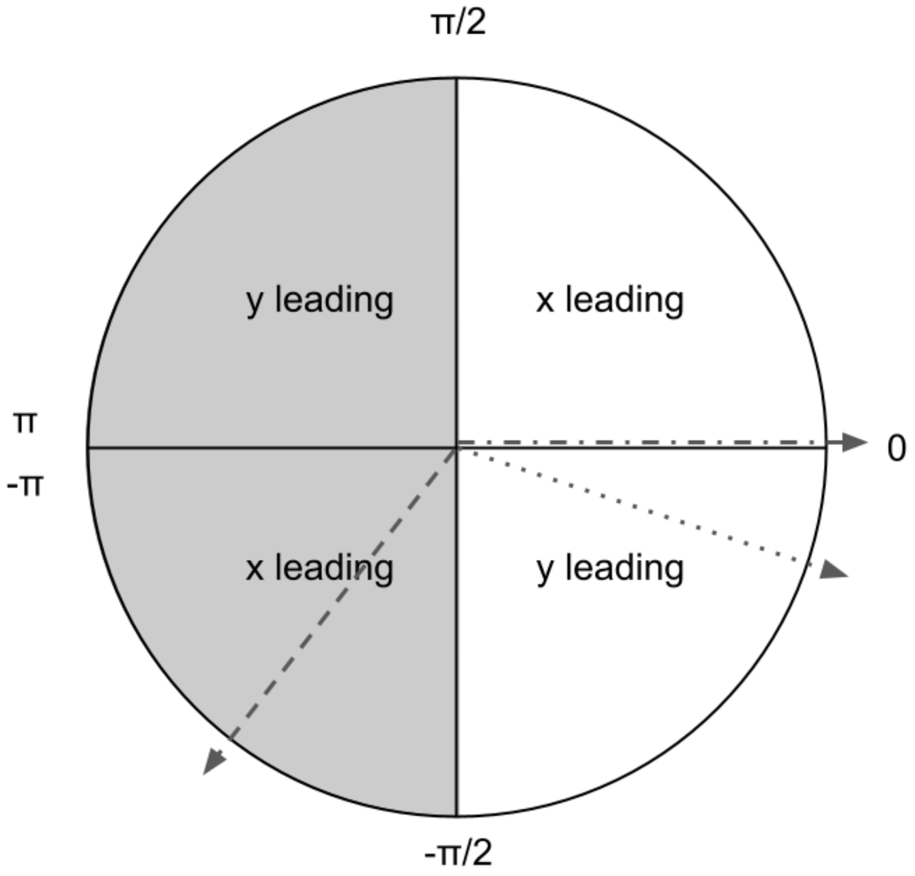

Here, Arg is the *argument* of the complex number, which results from the same calculation as for CWP shown in Eq. S3. The angle between the two dimensions can take on values on the interval $[-\pi, \pi]$, representing the difference between the two time series' phases, i.e., the leader-follower relationship in a dyad (see Aguiar-Conraria and Soares, 2011, for more details). Figure S1 represents the different possible values of $\mathrm{Phase}_{\mathrm{n}}^{\mathrm{j}}$ (adapted from Aguiar-Conraria and Soares, 2011; Rösch and Schmidbauer, 2018). Values of $\mathrm{Phase}_{\mathrm{n}}^{\mathrm{j}} \in (0, \frac{\pi}{2})$ indicate in-phase synchrony with partner 1 (x) leading before partner 2 (y). For $\mathrm{Phase}_{\mathrm{n}}^{\mathrm{j}} \in (0, -\frac{\pi}{2})$, partner y leads, within an in-phase synchronization scenario. If $\mathrm{Phase}_{\mathrm{n}}^{\mathrm{j}} \in (-\frac{\pi}{2}, -\pi)$ or $\mathrm{Phase}_{\mathrm{n}}^{\mathrm{j}} \in (\frac{\pi}{2}, \pi)$, synchronization is anti-phase, with x or y leading, respectively. For example, let us consider a case in which partner x is a parent and partner y is their child. When encountering the exemplary arrows shown in Figure S1, the dotted arrow would represent in-phase synchronization with the child leading. This may occur, for example, when the child initiates playing. The dashed arrow, i.e., anti-phase synchrony with the parent leading, may represent an instance where the child experiences emotional arousal, and the parent regulates this arousal by becoming calmer (Feldman, 2017). The dashed

and dotted arrow indicates in-phase synchrony with simultaneous changes in both parent and child. This could originate from a change in the environment to which both parent and child react in joint attention.

Phase difference information can be included in CWP plots (see Figures 5 and 6 in the manuscript). Information about in-phase and anti-phase synchrony and information about leader-follower relationships are thereby two different types of analyses that can be investigated separately or jointly. In this way, we can mitigate the drawbacks of CWP analysis of unspecific synchronization processes. In quantifying cross-wavelet-power-derived synchrony, we could differentiate between, e.g., in-phase and anti-phase values. Thus, researchers can take into account different leader-follower information to a larger or smaller degree when analyzing synchrony using CWP. However, compared to cross-correlation, this information is not as readily available and may not be included in some packages' implementation.

3.2 Example for phase difference analysis

To illustrate how phase differences may be used in synchrony analysis, we extracted phase-difference information from our simulated example data (Figures 5 and 6 in the manuscript). In the WaveletComp package (Rösch and Schmidbauer, 2025), phase differences are provided as a [frequency x time] matrix. Here, we investigated only those phase differences at time points and frequency values at which CWP coefficients were statistically significant (tested against white noise). The reasoning behind this is that leader-follower relationships become meaningless when no synchrony occurs because no adaptation to the other interaction partner emerges in the first place. In the next step, we categorized all statistically significant phase differences as in-phase or anti-phase, and as x leading or y leading. We defined edge cases in which no clear leadership occurred, i.e., for values around 0 and $\pm\pi$. We defined $0 \pm \frac{1}{36}$, $\pi \pm \frac{1}{36}$, and $-pi \pm \frac{1}{36}$ as cases where there was no lead but simultaneous changes. The resulting interpretation thus depends on the value range chosen to represent "no leadership". Table S1 shows the results for our simulated example data.

From this simple analysis of phase difference values for simulated example data, we can see the utility in differentiating between in-phase, anti-phase, and shifted synchrony types. For example, all significant phase differences for Figure 5A in the manuscript (in-phase synchrony) indicate in-phase synchrony, thus correctly detecting the synchrony type simulated in the time series. When the simulated data were not explicitly modeled to include a specific in-phase/anti-phase or leadership dynamic, the outcomes are more ambiguous. For example, in Figure 6B (change from synchrony in one frequency band to synchrony in two frequency bands), time series x leading, time series y leading, and no leadership occurred at approximately similar rates. In a real-life application, such a result would indicate that both interaction partners preceded the other in their changes in heart rate, but there was no overall leader of the interaction. However, changes over time could be analyzed, which could be connected to specific events over the course of the experiment.

With the information about the synchrony type, researchers may extract, e.g., only those CWP coefficients for which phase differences indicate in-phase synchrony. This may be relevant in situations where in-phase and anti-phase synchrony indicate differential processes (e.g., Reed et al., 2013). For example, average in-phase and anti-phase synchrony values, respectively, may indicate which synchrony type is (more) relevant in a given scenario. For example, our simulated data in Figure 5A, exemplifying in-phase synchrony data, have an average in-phase CWP coefficient (across all significant CWP values) of 0.60, whereas there were no significant anti-phase occurrences (i.e., we cannot calculate the anti-phase CWP average). This illustrates the potential use of dividing CWP coefficients into in-phase and anti-phase coefficients, which may be utilized in further analyses. For example, researchers could compare what happens during phases of in- and anti-phase synchrony, or what happens during phases where there is a leader compared to no

Table S1. Phase difference results for simulated data in Figures 5 and 6 in the manuscript.

Time series	Synchrony scenario	in-phase ratio [%]	x leading ratio [%]	y leading ratio [%]	no lead [%]
Figure 5 A	In-phase synchrony	100	26.44	19.60	53.96
Figure 5 B	Anti-phase synchrony	0	35.12	17.17	47.14
Figure 5 C	Shifted synchrony	0	0	100	0
Figure 5 D	No synchrony	37.07	56.94	42.12	0.94
Figure 6 A	Change from no synchrony to synchrony on three frequency bands	40.84	44.05	54.20	1.75
Figure 6 B	Change from synchrony in one frequency band to synchrony in two frequency bands	98.94	30.00	32.77	37.23
Figure 6 C	Change from synchrony in one frequency band to synchrony in another frequency band	100	39.43	27.27	33.20

lead. Thus, CWP allows for a fine-grained resolution permitting investigation of temporal and frequency changes, as well as interaction dynamics.

REFERENCES

Aguiar-Conraria, L. and Soares, M. J. (2011). *The Continuous Wavelet Transform: A Primer*. NIPE Working Papers 16/2011, NIPE - Universidade do Minho

Feldman, R. (2017). The neurobiology of human attachments 21, 80–99. doi:10.1016/j.tics.2016.11.007

Reed, R. G., Randall, A. K., Post, J. H., and Butler, E. A. (2013). Partner influence and in-phase versus anti-phase physiological linkage in romantic couples. *International Journal of Psychophysiology* 88, 309–316. doi:10.1016/j.ijpsycho.2012.08.009

Rösch, A. and Schmidbauer, H. (2018). WaveletComp 1.1: A guided tour through the R package

Rösch, A. and Schmidbauer, H. (2025). *WaveletComp: Computational Wavelet Analysis*. doi:10.32614/CRAN.package.WaveletComp. R package version 1.2

Veleda, D., Montagne, R., and Araujo, M. (2012). Cross-Wavelet Bias Corrected by Normalizing Scales. *Journal of Atmospheric and Oceanic Technology* 29, 1401–1408. doi:10.1175/JTECH-D-11-00140.1

3.3
